# Effects of the Maximum Recommended Levels of Fumonisins in the EU on Oxylipin Profiles in the Liver and Brain of Chickens

**DOI:** 10.3390/antiox14010019

**Published:** 2024-12-27

**Authors:** Philippe Guerre, Elodie Lassallette, Amélie Guerre, Didier Tardieu

**Affiliations:** IHAP, Université de Toulouse, INRAE, ENVT, 31076 Toulouse, France; elodie.lassallette@envt.fr (E.L.); amelie.guerre1@gmail.com (A.G.); didier.tardieu@envt.fr (D.T.)

**Keywords:** fumonisins, oxylipins, brain, liver, chicken

## Abstract

This study aimed to assess the effects of a diet containing 20.8 mg FB1 + FB2/kg over four and nine days on oxylipin (OL) profiles in the liver and brain of chickens. A total of 96 OLs, derived from seven polyunsaturated fatty acids (PUFAs) via the cyclooxygenase (COX), lipoxygenase (LOX), cytochrome P450 (P450), and non-enzymatic pathways, were measured using HPLC-MS/MS. In the liver, a significant increase in epoxide P450-derived OLs was detected by day 4, with smaller but notable increases in COX- and LOX-derived OLs by day 9. These alterations were independent of whether the parent PUFA was ω6 or ω3. However, OLs derived from 18-carbon (C18) PUFAs, such as linoleic acid and alpha-linolenic acid, showed greater increases compared to those derived from C20 or C22 PUFAs. The diol/epoxide ratios in the liver decreased at four and nine days, suggesting that fumonisins did not induce an inflammatory response. In the brain, at four days, the most discriminative OLs were derived from ω3-PUFAs, including docosahexaenoic acid, docosapentaenoic acid, and alpha-linolenic acid, via the LOX pathway. By nine days, several OLs derived from arachidonic acid, spanning all enzymatic pathways, became discriminative. In general, the diol/epoxide ratios in the brain were decreased at 4 days and then returned to the initial levels. Taken together, these results show strong effects of fumonisins on OLs in the liver and brain that are both specific and distinct.

## 1. Introduction

Fumonisins are mycotoxins primarily produced by molds of the *Fusarium* genus, with *F. verticillioides* (formerly *F. moniliform*) being the most prevalent producer. These toxins are found globally, mainly in corn and corn-derived products [1,2]. The B subfamily contains the most abundant and toxic fumonisins, with fumonisin B1 (FB1) being the most prevalent. The toxicological effects of fumonisins vary widely among species. In horses, they cause neurotoxicity, leading to equine leukoencephalomalacia [3]. In pigs, the lungs are the primary target, resulting in porcine pulmonary edema [4]. In most animal species, fumonisins induce liver or kidney toxicity, with liver damage being particularly pronounced in poultry and mice, while nephrotoxicity is more common in rats. Fumonisins are known to be hepatocarcinogenic in mice and nephrocarcinogenic in rats, and they are classified by the International Agency for Research on Cancer (IARC) as group 2B—possibly carcinogenic to humans [1,5]. A provisional maximum tolerable daily intake (PMTDI) for fumonisins B has been established for humans, and guidelines for maximum levels in animal feed have been set [1,6].

Fumonisins are known to induce a variety of cytotoxic effects, with the disruption of sphingolipid synthesis being the most extensively studied [7]. This effect stems from the structural similarity between FB1 and sphingoid bases, leading to numerous cellular alterations across multiple species. A hallmark of these changes is the significant variation in the sphingolipid profile observed in poultry [8]. While the primary effects of fumonisins on ceramide synthases (CerS) can explain many of the cellular changes [7], additional mechanisms of toxicity have been proposed [2,9,10,11,12]. These include altered apoptosis, changes in cell proliferation, oxidative stress, endoplasmic reticulum stress, mitochondrial dysfunction, and disruptions in membrane integrity [13]. Although it is challenging to discern whether these alterations are a direct cause or a consequence of fumonisin toxicity, it is clear that disturbances in lipid homeostasis are a consistent feature of fumonisin-induced cellular damage. Beyond sphingolipids, fumonisins also affect the levels of cholesterol, triglycerides, inositol phosphates, and polyunsaturated fatty acids (PUFAs) [13]. Paradoxically, no study to date has comprehensively examined the effects of fumonisins on the oxylipidome.

**Figure 1:**
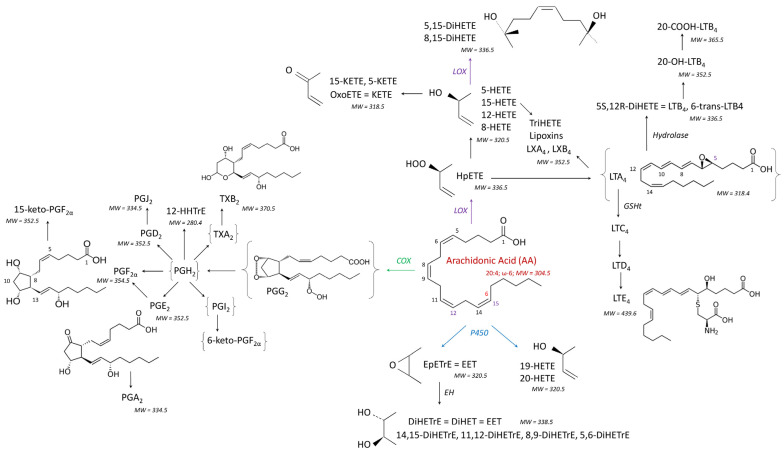
Main oxylipins formed from arachidonic acid via the COX, LOX, and P450 pathways, and after the action of EHs.

**Figure 2:**
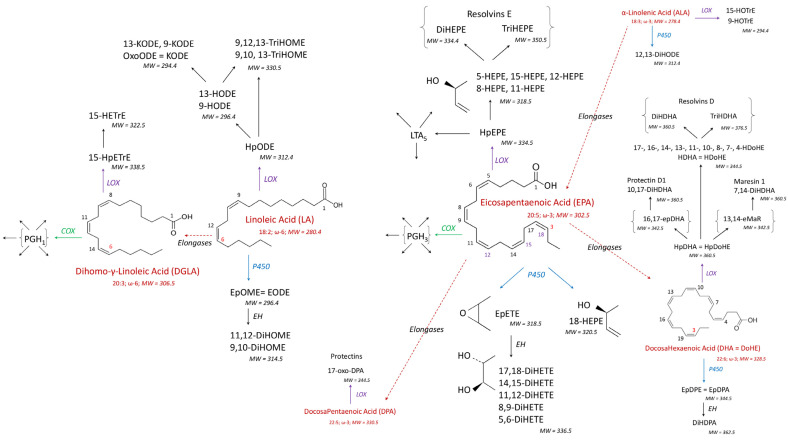
Main oxylipins formed from linoleic, dihomo-gamma-linoleic, alpha-linolenic, eicosapentaenoic, docosapentaenoic, and docosahexaenoic acids via the COX, LOX, and P450 pathways, and after the action of EHs.

Oxylipins (OLs) are bioactive lipids formed through the oxidation of PUFAs and play critical roles in various biological processes, with their functions varying depending on the specific PUFA precursor [14]. OLs derived from ω6-PUFAs, such as arachidonic acid (AA), linoleic acid (LA), gamma-linoleic acid (GLA), and dihomo-gamma-linoleic acid (DGLA) (Figure 1 and Figure 2), often have pro- or anti-inflammatory effects. In contrast, OLs produced from ω3-PUFAs like alpha-linoleic acid (ALA), eicosapentaenoic acid (EPA), docosapentaenoic acid (DPA), and docosahexaenoic acid (DHA) (Figure 2) are predominantly considered anti-inflammatory or neutral in nature [15,16].

The biological activity of OLs is also influenced by the metabolic pathway through which they are synthesized. OLs formed via the cyclooxygenase (COX) pathway from AA are mainly prostaglandins (PGs), which are typically pro-inflammatory. In contrast, OLs produced through the lipoxygenase (LOX) pathway can possess both pro- or anti-inflammatory properties (Figure 1). For example, 15-HpETE, a precursor to 15-HETE, exhibits both pro- and anti-inflammatory roles, while also serving as a precursor to lipoxins (Figure 2), which are specialized mediators involved in the resolution of inflammation [17]. Similarly, OLs derived from cytochromes P450 (P450) display diverse roles in inflammation. For instance, 20-HETE is pro-inflammatory, while EpETrE is anti-inflammatory. Their metabolites, DiHETrE, formed through the action of epoxide hydrolase (Figure 1), are pro-inflammatory, and the DiHETrE:EpETRE ratio increases during inflammatory responses [18,19,20,21]. Interestingly, while COX-mediated OLs from EPA are generally low in pro-inflammatory activity [14,22], the majority of OLs derived from EPA, DPA, and DHA through the LOX pathway are resolvins, protectins, and maresins (Figure 2), which are specialized mediators involved in the resolution of inflammation [17]. OLs can also be formed through the non-enzymatic (NE) oxidation of PUFAs, which typically occurs during oxidative stress within cells [23]. As a result, a variety of HPLC-MSMS techniques have been developed to assess OL variations in living organisms. These methods provide insights into OL content specific to particular PUFAs, metabolic pathways, or inflammatory roles [24,25,26,27,28].

The diverse effects of fumonisins, particularly their ability to reduce PUFA concentrations in cells [13], suggest that these toxins may disrupt the oxylipidome. Since a wide array of OLs with distinct biological functions are generated from various PUFAs within cells [14], a thorough examination of the oxylipidome—focusing on OLs derived from different PUFAs via multiple metabolic pathways—is highly warranted. To investigate this, we utilized an HPLC-MSMS method to profile 111 OLs, including 15 deuterated standards, originating from seven PUFAs through the COX, LOX, P450, and NE pathways (Figure 1 and Figure 2). Chickens were fed a diet containing 20.8 mg FB1 + FB2/kg for four and nine days with OL levels measured in both the liver and brain. This dose was chosen because it corresponds to the maximum of 20 mg FB1 + FB2 recommended by the EU in poultry feed [29]. An exposure period of 4 to 9 days was chosen to reveal the early effects of fumonisins. The liver was selected for analysis due to the accumulation of FB1 over time in this organ [30], which is the most sensitive to fumonisin-induced alterations in the sphingolipidome in chickens [8,31]. The brain was also studied, even though FB1 is not detectable in this organ, because fumonisins have been shown to alter the sphingolipidome in the brain in ways that cannot be fully explained by CerS inhibition alone [32].

## 2. Materials and Methods

### 2.1. Reagents and Chemicals

All reagents and analytes were sourced from Sharlab (Sharlab S.L., Sentmenat, Spain) or Sigma (Sigma-Aldrich Chimie SARL, Saint Quentin Fallavier, France). Solvents for oxylipin separation via HPLC-MSMS were of LC-MS grade, while all other reagents and solvents were HPLC grade. Indomethacin, triphenylphosphine, butylated hydroxytoluene, and AUDA were purchased from Sigma. OLs used as standards or internal standards (IS) were supplied by Interchim (Interchim, Montluçon, France) and correspond to Cayman chemical products (Cayman Chemical, Ann Arbor, MI, USA). The IS mixtures included the Primary COX and LOX MaxSpec^®^ LC-MS Mixture, containing 6-keto-prostaglandin F1α-d4, prostaglandin F2α-d4, prostaglandin E2-d4, prostaglandin D2-d4, thromboxane B2-d4, 15(S)-HETE-d8, 12(S)-HETE-d8, and (d8)5-HETE; the Deuterated Arachidonic Acid CYP450 Metabolite MaxSpec^®^ LC-MS Mixture, containing 20-HETE-d6, (±)14(15)-EET-d11, and (±)11(12)-EET-d11 each at a concentration of 1 µg/mL in ethanol; and the Deuterated Linoleic Acid Oxylipins MaxSpec^®^ LC-MS Mixture, containing (±)12(13)-DiHOME-d4, 13(S)-HODE-d4, 13-OxoODE-d3, and (±)12(13)-EpOME-d4 each at a concentration of 10 µg/mL in ethanol. Additional OL standards, as detailed in Table S1, were also sourced from the same supplier.

### 2.2. Fumonisin Diets and Sampling Protocol

The control diet, free from mycotoxins, and the fumonisin-containing diet were formulated using soya, wheat, and corn to meet the nutritional needs of the animals [30]. The fumonisin diet was prepared by incorporating maize naturally contaminated with fumonisins, achieving final concentrations of FB1, FB2, and FB3 of 15.2, 5.6, and 0.9 mg/kg, respectively. The mycotoxin levels in raw materials and diets were quantified by LC-MSMS according to the AFNOR standard V03-110 [33]. Among the 44 mycotoxins tested, only fumonisins were detected at significant levels [30].

This animal study was conducted at Cebiphar (Cebiphar, Fondettes, France), as detailed in [30], under project number V9152, project 2017062111426641, approved by the French Ministry of Higher Education, Research and Innovation on 6 November 2017. A total of 30 Ross broilers were randomly assigned into three groups of 10: one group receiving the mycotoxin-free control diet (Con), and two groups receiving the fumonisin-containing diet for four days (FB_4d), from day 17 to day 21, and for nine days (FB_9d), from day 12 to day 21. On day 21, the fumonisin diet was withdrawn eight hours prior to the sacrifice of the animals, and tissue samples were collected. Samples were stored at −80 °C until further analysis. No effects on performance or biochemical parameters were observed, as previously reported [30].

### 2.3. Preparation of Standards, Chromatographic Separation, and Mass Spectrometry

Stock solutions of OLs were prepared at a concentration of 10 µg/mL in ethanol and stored at −20 °C or −80 °C, following the manufacturer’s recommendations. Calibration solutions were prepared with concentrations of 1000, 500, 250, 125, 62.5, 31.25, 15.63, 7.81, 3.91, 1.95, 0.98, 0.49, 0.24, and 0.12 ng/mL in ethanol for all analytes, except for 13-HODE and 9-KODE, which were also prepared in concentrations of 20, 10, 5, 2.5, and 1.25 µg/mL in ethanol. These calibration solutions were freshly prepared before each use. A mixture of deuterated standards was also prepared (*v*/*v*/*v*) to serve as internal standards (ISs).

Chromatographic separation was performed using Poroshell 120 column (3.0 × 50 mm, 2.7 μm). Analytes were eluted using two mobile phases: (i) water/acetic acid (100:0.1, *v*/*v*, phase A); (ii) acetonitrile/isopropanol (90:10, *v*/*v*, phase B). Separation was conducted at a flow rate of 0.3 mL/min for 30 min at 40 °C with the following gradients: 0–3.5 min from 15% B to 33% B, 3.5–5 min to 38% B, 5–7 min to 42% B, 7–9 min to 48% B, 9–15 min to 65% B, 15–17 min to 75% B, 17–18.5 min to 85% B, 18.5–19.5 min to 95% B, 19.5–21 min to 15% B, and 21–30 min to 15% B [27]. Mass spectrometric detection was carried out using negative electrospray ionization under the following conditions: temperature 300 °C, flow rate of 10 L/min, pressure 25 psi, and capillary voltage 4000 V. Transitions, fragmentor voltages, and collision energies were optimized for each analyte using the Agilent MassHunter Optimizer software 2011 G3335-60091, with the results reported along with the retention times in Table S1. Data analysis was performed using Agilent MassHunter quantitative analysis software B.05.291.0. The accuracy of the method was considered acceptable, with a relative standard deviation (RSD) of less than 20%.

### 2.4. Stability, Linearity, and Limit of Quantitation

The stability of the standard solutions of OLs in ethanol was assessed at room temperature over periods of 8, 16, 24, and 32 h, with concentrations of 0.5, 2, 8, 31, 125, and 500 ng/mL (Table S2). Except for 5,6-EpETrE, which was deemed unstable, all other analytes remained stable for more than 24 h. The stability of 5-HpETE was 88% at 8 h and 65% at 24 h.

The linearity of the method was evaluated using OL solutions in ethanol at concentrations ranging from 0.1 to 1000 ng/mL, corresponding to 1 to 10,000 pg on column (Table S3). For 13-HODE and 9-KODE, to account for the high concentrations observed in the liver samples, the method was extended to concentrations of 10 and 20 µg/mL, respectively. Different concentration ranges were employed to address the variation in OL levels between the liver and brain (Table S3). The results demonstrated excellent linearity across all the OLs analyzed in this study, consistent with the previous findings [24,25,27,28]. The lowest concentration tested, which provided an accuracy of 80–120%, was determined to be the optimal limit of quantitation (LOQ).

### 2.5. Extraction and Purification of Samples

OLs in liver and brain tissues were determined using tissue homogenates prepared at 4 °C. For homogenization, 1 g of liver or brain tissue was combined with 3 mL of phosphate buffer (0.1 M, pH 7.4) and processed in a Teflon-coated Potter glass. The homogenate was then centrifuged at 3000× *g* for 15 min at 4 °C, and the supernatant (S3000) was collected and stored at −80 °C until analysis.

Liver and brain S3000 homogenates were extracted using 60 mg Oasis HLB 3cc extraction cartridges. Prior to extraction, the homogenates were diluted in NaCl buffer and mixed with an antioxidant cocktail following the methodology outlined in [24,25,27,28] with minor modifications. Specifically, 1850–1800 µL of 0.9% NaCl was added to 50–100 µL of the liver or brain S3000 homogenate. Then, 10 µL of the antioxidant cocktail containing EDTA (2 mg/mL), indomethacin (2 mg/mL), BHT (0.2 mg/mL), and triphenylphosphine (0.2 mg/mL) in a water/methanol/ethanol solution (2:1:1, *v*/*v*/*v*) was added, followed by 10 µL of AUDA (5 mg/mL in DMSO), 50 µL of 50% acetic acid, 170 µL of ethanol, and 30 µL of the IS mixture.

The mixture containing the OLs was passed over the columns under a maximum vacuum of 20 mm Hg. Following this, the columns were washed with 2 mL of 5% methanol and dried for 30 min. The OLs were subsequently eluted with 1 mL of methanol and 2 mL of ethyl acetate into a borosilicate glass tube containing 5 µL of glycerol/methanol (30:70, *v*/*v*). The eluate was dried at 40 °C, reconstituted in 200 µL of ethanol, centrifuged at 3000× *g* for 15 min, and filtered through 0.45 µm nylon membrane syringe filters before injection into the HPLC system.

Samples were processed in batches of twelve, each containing three control samples, three samples from chickens fed the fumonisins diet for four days, and three samples from chickens fed the fumonisins diet for nine days. The samples were injected alternately with a 10 µL volume, ensuring a maximum delay of 10 h between the thawing of tissue homogenates and the last injection into the HPLC system.

### 2.6. Recovery of Oxylipins in PBS

The recovery of OLs was assessed by spiking analytes into a solution of phosphate-buffered saline (PBS) and passing the spiked solution through the HLB columns, as described in the extraction and purification section. The concentrations tested ranged from 0.2 to 25 ng/mL PBS (0.1 M, pH 7.4), corresponding to 20 to 2500 pg spiked before extraction.

An acceptable recovery of 70 to 120%, accompanied by an RSD of less than 20%, was obtained for the majority of analytes tested (Table S4). A recovery of 60 to 70% was considered acceptable as long as the RSD was under 20%. Analytes with recoveries of less than 60% or greater than 130% across the 1–25 ng/mL concentration range and with an RSD of less than 20% were considered difficult to quantify. This included 11-trans-LTD4, 6-trans-LTB4, maresin 1, 14,15-DiHETE, 13-HODE, 9-HODE, and 16-HDoHE. These analytes were treated as providing comparative data between groups, but the quantities measured were regarded as estimates rather than precise values.

Any analyte with a mean RSD greater than 25% across the 1–25 ng/mL concentration range was deemed non-quantifiable in this study. This applied to 15-deoxy-δ12,14-PGD2, LTB4, and 5,6-EpETrE. Furthermore, 13-HpODE and 15-HpETE were not detected after passing through the HLB columns and were therefore considered non-quantifiable in this study.

### 2.7. Matrix Effect, Recovery, and Repeatability of Internal Standards in Liver and Brain

Signal suppression and enhancement (SSE) were evaluated for the IS by the method area at two concentrations in the liver and three concentrations in the brain (Table S5). SSE values outside the 80–120% range indicated a matrix effect, which was considered in the final concentration calculation. SSE at a concentration of 0.4 µg/g for the deuterated OLs from AA and 4 µg/g for the deuterated OLs from LA were deemed acceptable for all the analytes used as IS based on an RSD below 20%.

The recovery of IS on the HLB columns was measured for analytes spiked in PBS solution, as well as in liver and brain homogenates, as described in the extraction and purification section. A recovery of 70 to 120% was obtained in all three matrices for the majority of the IS (Table S6). The recovery of (d3)13-KODE in PBS, liver, and brain was 71%, 50%, and 57%, respectively. This recovery was consistent with the mean recovery of 13-KODE in PBS, which was 68% (Table S4). A very low recovery of (d8)15-HETE was observed in both PBS and tissues. Since the mean recovery of 15-HETE in PBS was 78% (Table S4), we decided not to use (d8)15-HETE as an IS. The concentrations of 15-HETE in tissues were corrected by the recovery measured for (d8)12-HETE.

Intra- and inter-day repeatability were assessed by the RSD of the recovery measured for the IS (Table S7). A value below 20% was considered acceptable.

### 2.8. Statistical Analysis

Statistical analyses were performed using XLSTAT Biomed 2018 software (Addinsoft, Bordeaux, France). To compare oxylipin concentrations in the liver and brain of chickens fed the control diet (n = 10) and the diet containing fumonisins for four (n = 10) or nine days (n = 10), a one-way ANOVA was performed after verifying homogeneity of variance using Hartley’s test. Differences among groups were considered statistically significant at *p* < 0.05. In cases where a significant difference was detected, individual means were compared using Duncan’s multiple range test. If variance homogeneity was not met, data were logarithmically transformed and re-analyzed. When variance remained non-homogeneous, the Kruskal–Wallis test was used. Statistically different means were indicated by different letters for the same variable.

The global effects of fumonisins on the oxylipidome were also evaluated using partial least squares discriminant analysis (PLS-DA). A Q2 value greater than 0.5 was considered indicative of a good predictive capacity for the model. Oxylipins with projection values greater than 1.1 were regarded as significant contributors to explaining the clustering of chickens into different groups.

## 3. Results

### 3.1. Effect of Fumonisins on Hepatic Oxylipidome

#### 3.1.1. Main Oxylipins Measured in Chicken Liver and Effect of Fumonisins

The OL levels in the livers of control chickens, categorized by the fatty acids and metabolic pathways responsible for their synthesis (Figure 1 and Figure 2), are presented in Table 1. A range of OLs derived from AA, EPA, DHA, LA, DGLA, and ALA were quantified in the liver. Among them, 38 OLs were formed from ω6-PUFAs, corresponding to AA, DGLA, and LA, and 26 OLs from ω3-PUFAs, specifically EPA, DHA, and ALA. These OLs are primarily produced via the COX, LOX, and P450 pathways. Additionally, a number of OLs are synthesized by the NE pathway, while some are metabolites formed from the action of enzymes, such as EHs, on other OLs. Among the OLs measured, those derived from LA and AA were the most abundant in chicken liver (Table 1).

**Figure 3:**
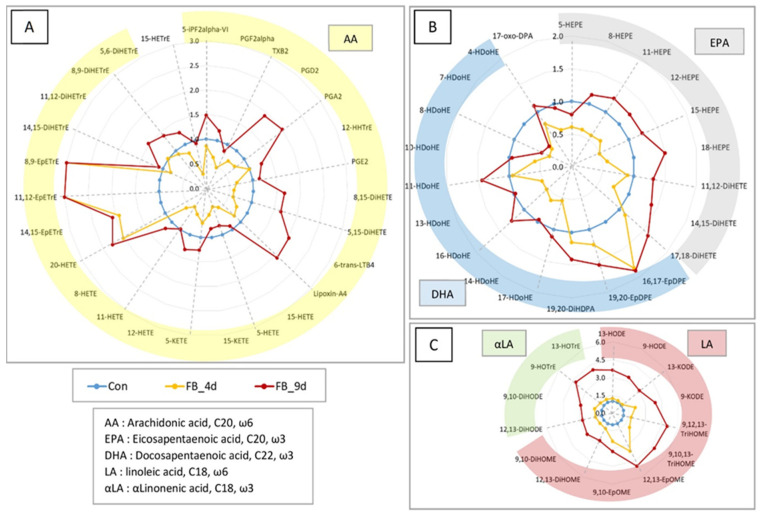
Effects of feeding chickens a diet containing 20.8 mg FB1 + FB2/kg for 4 days (FB_4d) and for 9 days (FB_9d) on oxylipin (OL) concentrations in the liver. Results are expressed as mean fold change in unexposed controls (Con) for 10 animals per group. (**A**) OLs from C20 polyunsaturated fatty acids (PUFAs) in ω6, corresponding to arachidonic acid (AA) and dihomo-gamma-linoleic acid. (**B**) OLs from C20-PUFAs in ω3, corresponding to docosahexaenoic acid and docosapentaenoic acid, and from C22-PUFAs in ω3, eicosapentaenoic acid. (**C**) OLs from C20-PUFAs, ω6-unsaturated linoleic acid, and ω3-unsaturated alpha-linolenic acid.

The effects of fumonisins administered for four and nine days at a dose of 20.8 mg FB1 + FB2/kg feed on hepatic oxylipidome are also presented in Table 1. At nine days of exposure, a significant increase in the levels of OLs derived from AA and a small number of OLs derived from EPA and DHA was observed. In contrast, at four days, the effects of the fumonisins were more variable, with some OL levels decreased and others increased. Regarding the OLs derived from LA and ALA, all were strongly elevated at nine days, while at four days, some OLs showed a weak increase, and others did not differ from the control levels. The role of the specific metabolic pathway involved in the synthesis of these OLs, as well as the influence of ω6- or ω3-unsaturation, remains complex. To further explore these effects, a complementary analysis was conducted, presenting the levels of OLs in the liver of fumonisin-fed chickens as fold changes relative to the control values (Figure 3).

#### 3.1.2. Effect of Fumonisins on OL Levels Expressed as Fold Change

At four days, the OLs from AA via the COX and LOX pathways remained unaffected or even tended to decrease (Figure 3A). In contrast, the OLs formed by the P450 pathway, such as 20-HETE, 14,15-EpETRE, 11,12-EpETRE, and 8,9-EpETRE, were strongly increased. At nine days, some OLs produced from the COX pathway—like PGD2 and PGA2—were elevated, while others, such as TXB2 and PGE2, were unaffected. Similarly, at nine days, various OLs derived from the LOX pathway, such as 8,15-DiHETE, 5,15-DiHETE, 6-trans LTB4, and lipoxin A4, were increased, while OLs like 5-HETE, 15-HETE, 12-HETE, and their metabolites 5-KETE and 15-KETE remained unaffected. For OLs from the P450 pathway at nine days, the 20-HETE, 14,15-EpETrE, 11,12-EpETrE, and 8,9-EpETrE levels were close to those measured at four days, but the EH metabolites of EpETrE, such as 14,15-DiETrE, 11,12-DiETrE, 8,9-DiETrE, and 5,6-DiETrE, showed an increase (Figure 3A).

The same analysis was performed for OLs derived from EPA and DHA (Figure 3B). At four days, only 16,17-EpDPE and 19,20-EpDPE, both produced by the P450 pathway, were elevated. At nine days, the effects of fumonisins on EpDPE were similar to those observed at four days. Moreover, 19,20-DiHDPA (produced by EHs on 19,20-EpDPE), along with 17,18-DiHETE, 14,15-DiHETE, and 11,12-DiHETE (produced by EHs on EpETEs), were also increased. It is also noteworthy that 18-HEPE, synthesized by the P450 pathway, showed a greater increase compared to 8-, 11-, 12-, and 15-HEPE, which were produced by the LOX or NE pathways. In general, LOX- and NE-derived OLs from EPA and DHA were reduced at four days but returned to control levels at nine days (Figure 3B).

For OLs from LA and ALA, 12,13-EpOME and 9,10-EpOME—produced by the P450 pathway—were strongly elevated in chickens fed fumonisins for four days (Figure 3C). OLs like 9,10,13-TriHOME, 9,12,13-TriHOME, and 9-KODE, formed via the LOX pathway (Figure 2), also increased. Other LA- and ALA-derived OLs showed no significant change at four days. By nine days, all LA- and ALA-derived OLs were elevated, regardless of the metabolic pathway responsible for their formation (Figure 3C).

In summary, the analysis of the effects of fumonisins on hepatic oxylipidome in chickens fed fumonisins for four days revealed a sharp increase in the OLs produced by the P450 pathway, while the OLs formed via the COX, LOX, and NE pathways remained unaffected or even decreased. OLs from AA, EPA, and DHA tended to decrease, whereas OLs from LA and ALA were unaffected. For chickens fed fumonisins for nine days, OLs from the P450 pathway were at concentrations close to those measured at four days, but their EH metabolites were elevated. The effect of fumonisins on OLs from AA, EPA, and DHA, which were formed via the COX, LOX, and NE pathways, varied. By contrast, all OLs from LA and ALA showed a significant increase. Given that the effects of fumonisins varied according to the duration of exposure, fatty acid, and metabolic pathway, partial least squares discriminant analyses (PLS-DA) were conducted to identify the most important variables in these effects.

#### 3.1.3. Main OLs for Discriminating the Effects of Fumonisins

**Figure 4:**
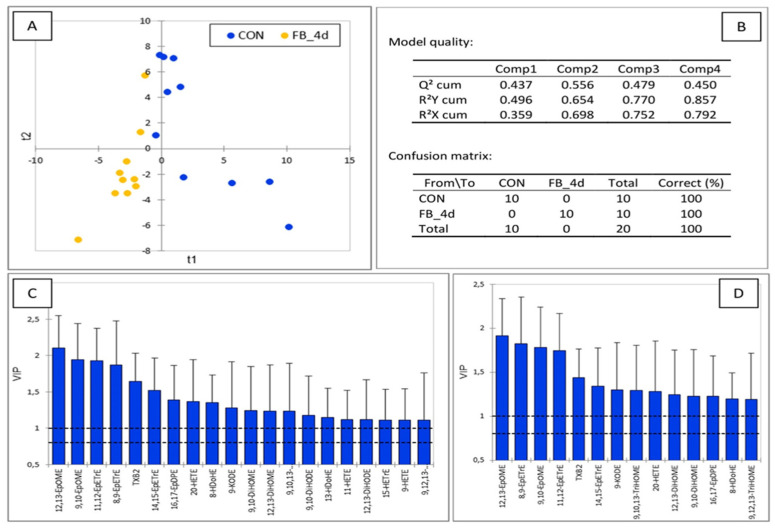
Partial least square discriminant analysis of oxylipins in the liver of chickens fed a control diet free of mycotoxins and chickens fed a diet containing 20.8 mg FB1 + FB2/kg for 4 days. (**A**) Discrimination on the factor axes extracted from the original explanatory variables. (**B**) Quality of the model and confusion matrix. Variables important in the projection for the first (**C**) and second (**D**) components.

At four days of exposure, the PLS-DA model provided excellent discrimination between control and fumonisin-fed animals, as demonstrated by the 100% sensitivity and specificity values (Figure 4). The Q2 value of 0.556, calculated on the first two components, indicated the robustness of the model. The main discriminant OLs on both axes corresponded to EpOME, EpETrE, and EpDPE, which are formed by the P450 pathway from LA, AA, and EPA (Figure 1 and Figure 2). Additionally, 20-HETE, also formed by P450, along with DiHOME and DiHODE, formed by the action of EHs on EpOME and EPODE, were also discriminating factors. Other OLs appearing as variables important in the projection (VIP) were of lesser importance, being synthesized from various PUFAs via the COX, LOX, and NE pathways.

**Figure 5:**
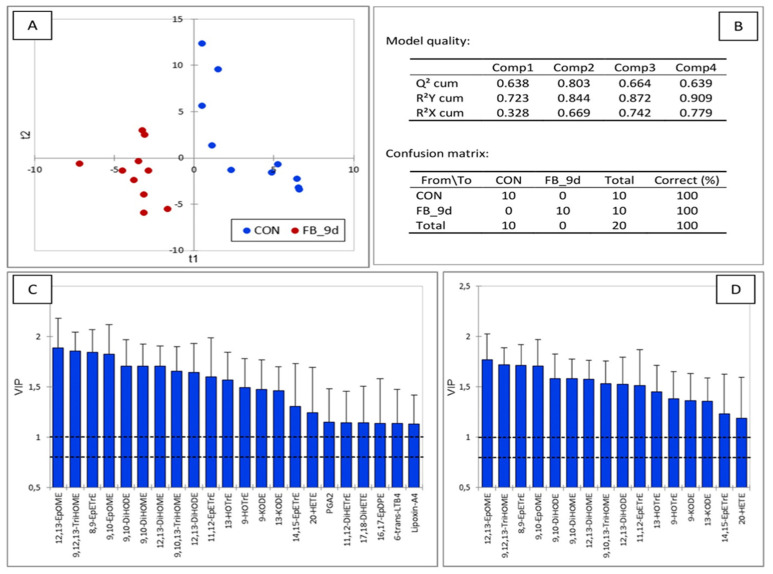
Partial least square discriminant analysis of oxylipins in the liver of chickens fed a control diet free of mycotoxins and chickens fed a diet containing 20.8 mg FB1 + FB2/kg for 9 days. (**A**) Discrimination on the factor axes extracted from the original explanatory variables. (**B**) Quality of the model and confusion matrix. Variables important in the projection for the first (**C**) and second (**D**) components.

The PLS-DA performed on the OL levels in chickens fed fumonisins for nine days, compared to the controls, revealed a very robust model, with a Q2 value of 0.803 (Figure 5). The main discriminant OLs on both axes corresponded to EpOME, EpETrE, EpDPE, and 20-HETE, formed by the action of P450 on LA, AA, EPA, and AA. DiHODE and DiHOME, along with 11,12-DiHETrE and 17,18-DiHETE, formed by the action of EHs on EPODE, EpOME, 11,12-EpETrE, and 17,18-EpETE, were also important in the model. Other OLs appearing as VIP were formed from various PUFAs by the COX, LOX, and NE pathways. Notably, a large number of discriminating OLs, such as 9,10,13-TriHOME, 9,12,13-TriHOME, 9-KODE, and 13-KODE, were from LA, and from ALA, OLs such as 9-HOTrE and 13-HOTrE were also significant.

Thus, PLS-DA revealed that the main discriminating variables in the effects of fumonisins on hepatic oxylipidome, at both four and nine days of exposure, were OLs produced by the P450 pathway. OLs from LA and ALA via the LOX pathway were also highly discriminating at nine days.

#### 3.1.4. Effect of Fumonisins on Diol/Epoxide Ratios

**Figure 6:**
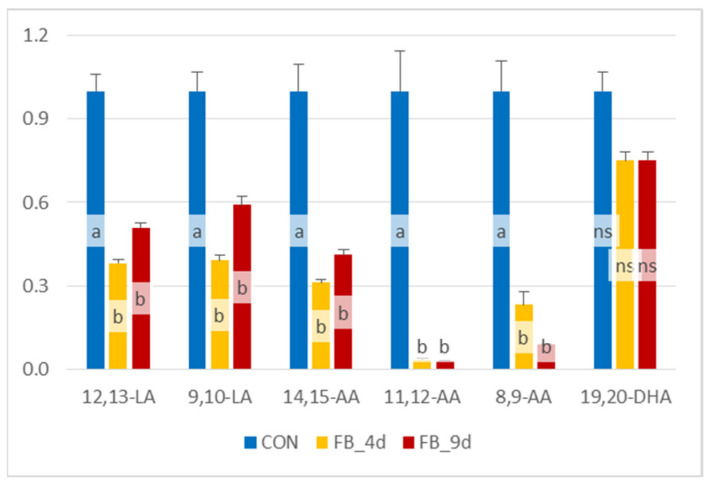
Diol/epoxide ratios calculated for oxylipins obtained from linoleic acid (LA), arachidonic acid (AA), and docosahexaenoic acid (DHA) in the liver of chickens fed a control diet free of mycotoxins (CONs) and chickens fed a diet containing 20.8 mg FB1 + FB2/kg for 4 days (FB_4d) or for 9 days (FB_9d). 9,10-LA and 12,13-LA corresponded to DiHOME:EpOME ratios; 14,15-AA, 11,12-AA and 8,9-AA corresponded to DiHETrE:EpETrE ratios; 19,20-DHA corresponded to 19,20-DiHDPA:19,20-EpDPE ratio. Values are expressed as mean ± SE, n = 10 per group. ANOVA was used to assess differences between groups. Statistically different groups (Duncan) are identified by different letters (*p* < 0.05).

The diol/epoxide ratios were calculated for different OLs derived from LA, AA, and DHA in the controls and chickens fed fumonisins for four and nine days (Figure 6). The 12,13-DiHOME:12,13-EpOME and 9,10-DiHOME:9,10-EpOME ratios, calculated for LA metabolites, were significantly reduced after four days of fumonisin exposure. After nine days of fumonisin feeding, these ratios remained similar to those measured at four days. The 14,15-DiHETrE:14,15-EpETrE, 11,12-DiHETrE:11,12-EpETrE, and 8,9-DiHETrE:8,9-EpETrE ratios for OLs derived from AA followed the same trend observed for LA metabolites. Meanwhile, the 19,20-DiHDPA:19,20-EpDPE ratio showed a slight decrease in chickens fed fumonisins, although this variation was not statistically significant.

### 3.2. Effect of Fumonisins on Brain Oxylipidome

#### 3.2.1. Main Oxylipins Analyzed in Chicken Brain and the Impact of Fumonisins

**Figure 7:**
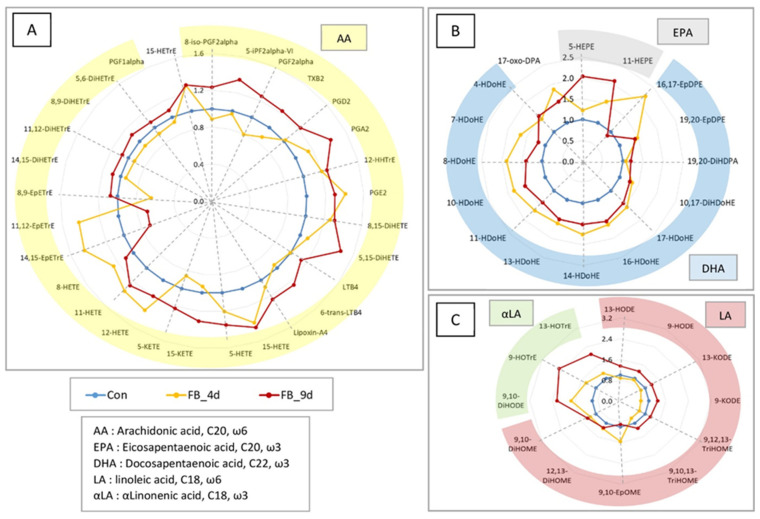
Effects of feeding chickens with a diet containing 20.8 mg FB1 + FB2/kg for 4 days (FB_4d) and for 9 days (FB_9d) on oxylipin (OL) concentrations in the brain. Results corresponded to the mean values obtained for 10 animals per group expressed as fold change in unexposed controls (Con). (**A**) OLs from C20 polyunsaturated fatty acids (PUFAs) in ω6, corresponding to arachidonic acid (AA) and dihomo-gamma-linoleic acid. (**B**) OLs from C20-PUFAs in ω3, corresponding to docosahexaenoic acid DHA and docosapentaenoic acid, and from C22-PUFAs in ω3, eicosapentaenoic acid (EPA). (**C**) OLs from C20-PUFAs, ω6-unsaturated linoleic acid (LA), and ω3-unsaturated alpha-linolenic acid (ALA).

In this study, 39 OLs derived from ω6-PUFAs and 19 from ω3-PUFAs were quantified in chicken brains, including metabolites from AA, EPA, DHA, LA, DGLA, and ALA (Table 2). These OLs were produced through the COX, LOX, P450, and NE pathways. Additionally, metabolites generated via EH activity were identified in brain tissue. The impact of fumonisins administered for four and nine days at a dose of 20.8 mg FB1 + FB2/kg feed was assessed, with the results presented in Table 2 (concentration data) and Figure 7 (fold change relative to unexposed controls).

After four days of fumonisin exposure, most AA-derived OLs exhibited no significant alterations (Figure 7A). Among those that differed, no consistent effects tied to a specific metabolic pathway were evident. However, after nine days of exposure, COX-derived OLs from AA, such as PGF2α, TXB2, and 12-HHTrE, showed significant increases. While LOX-derived OLs from AA also trended upward at nine days, these changes did not reach statistical significance. The effects of P450-derived OLs from AA were less consistent, with 8,9-EpETrE decreasing at four days and increasing at nine days (Figure 7A).

In contrast, most OLs from DPA and DHA exhibited significant increases as early as four days of fumonisin exposure. These elevations persisted through nine days (Table 2, Figure 7B). OLs derived from LA remained largely unaffected by fumonisin exposure. However, ALA-derived OLs showed a tendency to increase after nine days, although these changes were not statistically significant (Table 2, Figure 7C).

Overall, fumonisins exerted the most pronounced effects on OLs derived from DPA, DHA, and, to a lesser extent, EPA. The COX, LOX, and NE pathways were the primary contributors to the formation of these altered OLs. Given the relatively small variations in OL concentrations, PLS-DA was employed to determine whether fumonisin exposure could effectively differentiate treatment groups and identify the key OLs driving this separation.

#### 3.2.2. Discrimination of Chickens into Different Groups

**Figure 8:**
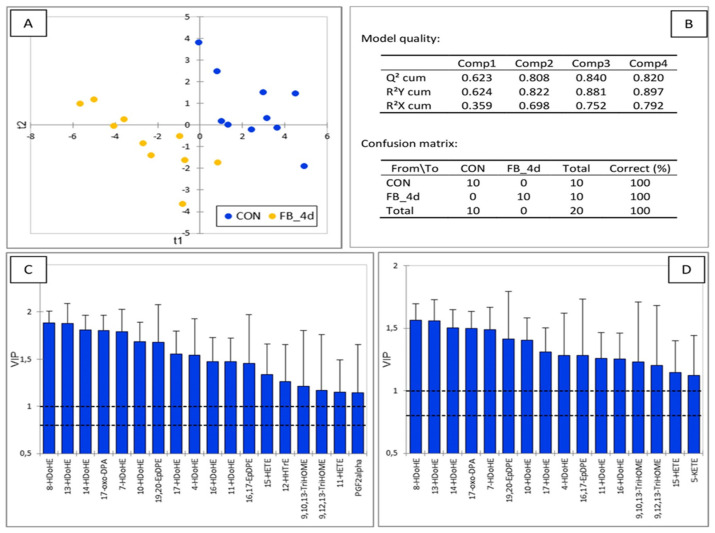
Partial least square discriminant analysis of oxylipins in the brain of chickens fed a control diet free of mycotoxins and chickens fed a diet containing 20.8 mg FB1 + FB2/kg for 4 days. (**A**) Discrimination on the factor axes extracted from the original explanatory variables. (**B**) Quality of the model and confusion matrix. Variables important in the projection for the first (**C**) and second (**D**) components.

The PLS-DA conducted on OLs measured in the controls and in chickens fed fumonisins for four days revealed a clear and distinct separation between the two groups (Figure 8). The robustness of the model was evidenced by a Q2 value of 0.808. The primary OLs responsible for this separation were derived from DHA. In addition, 17-oxo-DPA, derived from DPA, and 9,12,13-TriHOME and 9,10,13-TriHOME, derived from ALA, contributed significantly to the differentiation on the first two components. Only a small number of OLs from other fatty acids were identified as discriminating factors.

**Figure 9:**
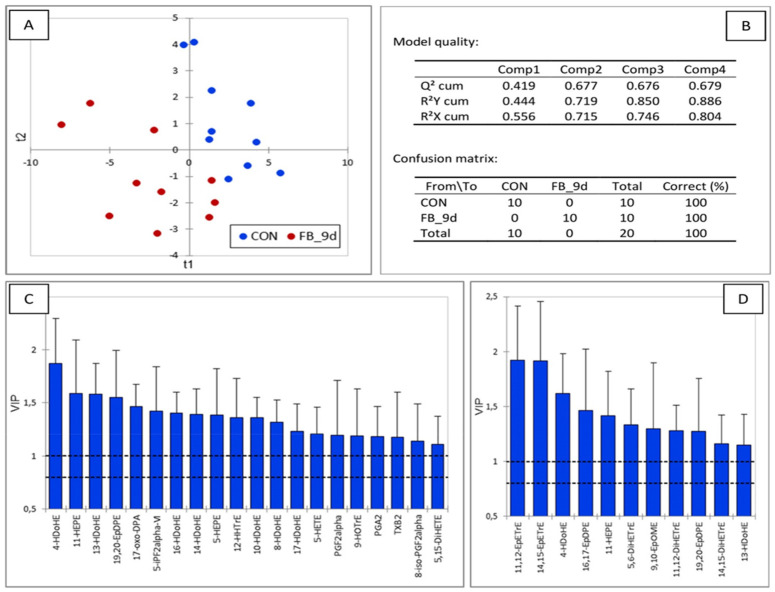
Partial least square discriminant analysis of oxylipins in the brain of chickens fed a control diet free of mycotoxins and chickens fed a diet containing 20.8 mg FB1 + FB2/kg for 9 days. (**A**) Discrimination on the factor axes extracted from the original explanatory variables. (**B**) Quality of the model and confusion matrix. Variables important in the projection for the first (**C**) and second (**D**) components.

After nine days of fumonisin feeding, the PLS-DA model again provided excellent separation between the controls and exposed chickens (Figure 9). The Q2 value of 0.677, calculated from the first two components, confirmed the model’s robustness. On the first component, the primary discriminating OLs were derived from DHA, along with a few OLs from AA formed via the COX pathway. On the second component, the main discriminating OLs were derived from AA via the P450 pathway, with some additional contributions from DHA-derived OLs.

In summary, the PLS-DA analysis of brain OLs allowed for a clear discrimination between fumonisin-fed chickens and unexposed controls. At four days, the differentiation was primarily driven by OLs derived from ω3-PUFAs. By nine days, the discriminating OLs included those derived from ω3-PUFAs as well as OLs formed from AA via the P450 pathway.

#### 3.2.3. Effect of Fumonisins on Diol/Epoxide Ratios

**Figure 10:**
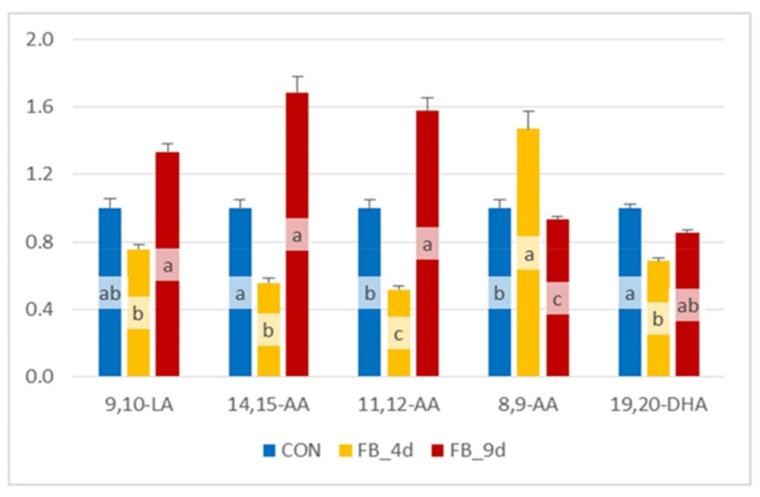
Diol/epoxide ratios calculated for oxylipins obtained from linoleic acid (LA), arachidonic acid (AA), and docosahexaenoic acid (DHA) in the brain of chickens fed a control diet free of mycotoxins (Con) and chickens fed a diet containing 20.8 mg FB1 + FB2/kg for 4 days (FB_4d) or for 9 days (FB_9d). 9,10-LA corresponded to 9,10-DiHOME:9,10-EpOME ratio; 14,15-AA, 11,12-AA and 8,9-AA corresponded to DiHETrE:EpETrE ratio; 19,20-DHA corresponded to 19,20-DiHDPA:19,20-EpDPE ratio. Values are expressed as mean ± SE, n = 10 per group. ANOVA was used to assess differences between groups. Statistically different groups (Duncan) are identified by different letters (*p* < 0.05).

The effects of fumonisins on the diol/epoxide ratios are presented in Figure 10. Ratios such as 9,10-DiHOME:9,10-EpOME, 14,15-DiHETrE:14,15-EpETrE, 11,12-DiHETrE:11,12-EpETrE, and 19,20-DiHDPA:19,20-EpDPE decreased in chickens fed fumonisins for four days. However, in chickens exposed to fumonisins for nine days, these ratios tended to return to values similar to those measured in the controls. Interestingly, the 8,9-DiHETrE:8,9-EpETrE ratio appeared to behave differently, increasing after four days of exposure and showing a tendency to decrease after nine days.

## 4. Discussion

### 4.1. Effect of Fumonisins on Hepatic Oxylipidome

The administration of a diet containing 20.8 mg FB1 + FB2/kg for nine days increased the levels of a large number of OLs in the liver. Given that this is the first study to explore the effects of fumonisins on oxylipidome, direct comparisons to previous research are difficult. However, it is noteworthy that the elevated OL concentrations found in the liver align with the findings from other studies showing decreased PUFA concentrations [13]. Since OLs are produced by the oxidation of PUFAs and fumonisins are known to induce oxidative stress [13], one might hypothesize that oxidative stress in the liver could explain the observed increase in OLs. However, this hypothesis seems unlikely in the context of this study. The effects of fumonisins on OLs were similar for both ω3- and ω6-PUFAs (Figure 3), despite the fact that ω3-PUFAs are more susceptible to oxidation than ω6-PUFAs [13,14,17]. While there are some exceptions, this observation is important because it is widely accepted that oxylipins derived from ω3-PUFAs generally exhibit anti-inflammatory effects, whereas those derived from ω6-PUFAs exhibit both pro- and anti-inflammatory roles [14,17,22]. Moreover, OLs produced through the NE pathway were less elevated in this study compared to those generated via enzymatic pathways, further questioning the likelihood of a nonspecific increase in OLs due to oxidative stress. Additionally, although P450-derived epoxides and their diol metabolites were generally increased, the diol/epoxide ratios for OLs derived from LA, AA, and DHA were reduced in chickens fed the fumonisin diet for four and nine days, as compared to the controls (Figure 6). This result is particularly significant because epoxide derivatives of PUFAs, such as EpETrE, are considered anti-inflammatory, while diol metabolites, such as DiHETrE, are regarded as pro-inflammatory [17]. The diol/epoxide ratios provide a measure of the balance between pro- and anti-inflammatory processes, with an increase in these ratios commonly observed during liver injury in alcohol-associated hepatitis [20]. Thus, the observed reduction in diol/epoxide ratios further challenges the hypothesis of a substantial oxidative stress response in the liver of chickens fed fumonisins. Although some studies have reported oxidative damage in fumonisins-fed chickens and laying hens [13], the results presented here align with multiple studies in poultry, where fumonisin doses around 20 mg FB1 + FB2/kg in feed did not increased MDA and GSH concentrations, as well as the activities of antioxidant enzymes like catalase, superoxide dismutase, glutathione peroxidase, and glutathione reductase [34,35,36].

A second hypothesis to explain the effects of fumonisins on liver OLs is that fumonisins may induce the enzymes responsible for OL synthesis. This hypothesis is supported by the observation that the OLs most increased in chickens fed fumonisins for nine days were those produced by the P450 pathway, with a smaller increase observed in those produced by the LOX pathway (Figure 3). This finding is even more striking in chickens fed fumonisins for just four days, as the only OLs showing increases were those produced by the P450 pathway, while other OLs either remained unchanged or decreased. Previous studies have suggested that fumonisins can increase P450 expression and activity in the liver, digestive tract, and in ex vivo systems [37,38,39]. However, contrasting findings have also been reported. For instance, a decrease in P450 activity, accompanied by endoplasmic reticulum damage, was observed in quails fed a diet containing 30 mg FB1/kg [40]. This study in chickens further revealed that fumonisin administration did not significantly alter the levels of COX-derived OLs in the liver. In contrast, a study on pig explants demonstrated that FB1 increased COX2 expression and induced oxidative stress [41]. The differences between these studies may be attributed to the concentrations of fumonisins used. The pig explant study employed a concentration of 70 µmol FB1/L [41], whereas the FB1 concentration measured in the liver of chickens in this study was much lower, at 0.044 µmol/kg [30].

In this study, the impact of fumonisins on the liver oxylipidome was influenced by the size of the PUFA parent. Specifically, OLs derived from C18-PUFAs were more increased compared to those from C20- or C22-PUFAs (Figure 3). This chain length-specific effect is consistent with previous research on other lipid classes. For example, fumonisins have been shown to elevate the C22–24:C16 ratios of sphingolipids, which have been proposed as a potential biomarker in poultry [8,31,32,42,43,44]. A similar pattern of variable effects, depending on fatty acid chain length and target tissue, has been observed in pigs for sphingolipids [45] and across species for other lipid classes [13]. Several hypotheses, not mutually exclusive, can explain these fatty acid chain length-dependent effects of fumonisins. First, the varying effects may be attributed to differences in fumonisins’ affinity for the catalytic sites of enzymes involved in lipid metabolism. This is particularly relevant for CerS at lower FB1 concentrations, with higher concentrations potentially leading to non-selective inhibition [7,42]. Second, the inhibition of key enzymes in lipid metabolism could trigger compensatory lipid synthesis, with the level and specificity of such compensatory mechanisms varying across different tissues [45,46,47]. Third, fumonisins, or the initial changes in the lipidome they induce, could modulate the activity of elongases. Elongases are crucial enzymes involved in the formation of very-long-chain fatty acids, with seven distinct types, each with specificities for different fatty acid chains [48]. ELOV1 to ELOV5 are responsible for elongating C18 to C26 fatty acids, while ELOV6 is involved in the elongation of C12 to C16 fatty acids [48]. In chickens, a large proportion of LA and ALA is supplied directly by the feed, while C20- and C22-PUFAs are in part synthesized in the liver [49,50]. Therefore, fumonisins’ potential impact on elongase activities could explain the more pronounced effects on LA- and ALA-derived OLs compared to AA-, EPA-, and DHA-derived OLs. The idea that fumonisins modulate elongase activity has been previously proposed to explain their toxicity in X receptor-deficient mice [51].

### 4.2. Effect of Fumonisins on the Cerebral Oxylipidome

In this study, the concentrations of OLs derived from LA in chicken brains were less abundant than those derived from AA and DHA. This finding aligns with the relative abundance of these PUFAs, as AA and DHA make up approximately 25% of fatty acids in the mammalian brain [19,52]. Although the quantitative effects of fumonisins on cerebral OLs were less pronounced compared to those observed in the liver, their qualitative impact was strong, as indicated by the Q2 values obtained from the PLS-DA analysis. The primary discriminating OLs after four days of feeding the fumonisins-containing diet were derived from DPA, DHA, and ALA through both the COX and LOX pathways. Given that DPA, DHA, and ALA are ω3-unsaturated PUFAs, this suggests the occurrence of an inflammatory/anti-inflammatory balance in the brain. Specifically, 17-oxo-DPA, generated by COX-2 during inflammation in macrophages, modulates the NF-κB signaling pathway, inhibiting the production of pro-inflammatory cytokines and nitric oxide, thereby exerting anti-inflammatory effects [53]. Moreover, LOX derived from 14-DHDoHE and 17-HDOHE, which are involved in the formation of protectins, resolvins and maresins (Figure 2), is a key player in resolving inflammation [17]. Most of the DHA-derived OLs are known to counteract inflammation in the brain, with some being produced by LOX and others by auto-oxidation [54]. Additionally, TriHOME compounds, such as 9,10,13-TriHOME and 9,12,13-TriHOME, were also significant discriminators in the brain in this study. A large number of stereoisomers of 9,10,13-TriHOME and 9,12,13-TriHOME have been described in humans [55]. These compounds, which can be synthesized by 15-LOX from LA, have been shown to play a role in neuronal morphogenesis [17]. Another discriminatory OL was 15-HETE, produced by 15-LOX from AA, which strongly increased in the brain after four days of fumonisins administration. While 15-HETE is often considered a pro-inflammatory OL, it also has direct anti-inflammatory properties and can be metabolized into lipoxins (Figure 1), which are crucial anti-inflammatory OLs derived from AA [14]. Taken together, these findings suggest that as early as four days after exposure to fumonisins, an inflammatory/anti-inflammatory response may be occurring in chicken brains.

After feeding the fumonisin-containing diet for nine days, the PLS-DA model still provided excellent separation between the control group and the chickens exposed to fumonisins (Figure 9). The main discriminating OLs at nine days were those identified at four days, along with a few additional ones. Among these, 9-HOTrE derived from ALA via the LOX pathway was the most increased and discriminated OL at nine days. Other discriminating OLs included 11,12-EpETrE, 14,15-EpETrE, 16,17-EpDPE, 19,20-EpDPE, and 9,10-EpOME, which are produced by the P450 pathway, along with their metabolites formed through the action of EHs. Interestingly, while the ratio of 8,9-DiHETrE to 8,9-EpETrE was increased at four days in chickens fed the fumonisin diet, most of the diol/epoxide ratios were decreased at four days and increased after nine days of fumonisin feeding. This shift in ratios is noteworthy, as elevated diol/epoxide ratios have been reported in the brain of rats exposed to neurotoxic compounds and during neurodegenerative processes [19,21].

Taken together, these results indicate the onset of an inflammatory/anti-inflammatory process in the brain, emerging as early as four days after exposure to the fumonisins-containing diet and intensifying by nine days. The alterations observed in the oxylipidome suggest that the primary OLs affected by the diet are those derived from the LOX and COX pathways, indicating that the impact of fumonisins on OLs is not merely the result of nonspecific oxidative stress. As fumonisins were undetectable in the brain at four and nine days [32], their effects on OLs may stem from interactions with glial cells or immune system cells. This hypothesis is consistent with prior studies suggesting that fumonisins’ neurotoxicity is linked to their effects on glial cells [56]. Furthermore, this aligns with previous findings on fumonisins’ influence on sphingolipids levels in the brain, alterations which cannot be solely attributed to the inhibition of CerS [32]. The changes in OLs in the brain could thus result from the early effects of fumonisins on sphingolipid metabolism [7], as d18:1P and ceramides, which are elevated in the brains of chickens fed fumonisins, are known to activate phospholipase A2 and promote the release of PUFAs in cells [57,58]. Further studies are necessary to validate this hypothesis.

### 4.3. Future Research Directions

The results of this study suggest that the effects of fumonisins on OLs are specific and different in the liver and brain. A comparison of the results observed at 4 and 9 days in the liver shows that the effects of fumonisins increased with the duration of exposure and mainly corresponded to an increase in OLs produced by the P450 system. As previous studies have shown that fumonisins induce P450 [37,38,39], and FB1 accumulates with time in the liver [30], an analysis of the expression of P450 could reinforce this observation.

The effects of fumonisins on OLs in the brain are more complex and are observed, while no FB1 can be detected [32]. An effect of fumonisins on sphingolipids suggestive of neuroinflammation, which cannot be explained by ceramide synthase inhibition alone, was also reported in the brain [32]. Taken together, these results suggest that a joint study of OLs and sphingolipids may not only confirm the effects of fumonisins but also reveal possible lipids interactions. Although seldom studied, the relationships between OLs and sphingolipids appear to be important in brain diseases [17]. A future study could also include the measurement of cytokines, which play a key role in the inflammatory response. Analyses at the protein level should also show whether the effects observed at the lipid level are associated with variations in enzyme expression and activity.

Further work on glial cells, neurons, and brain organoids could reveal cell interactions [59,60]. These models also allow for the effects of enzyme inhibitors or inducers to be studied. The lack of the effect of fumonisins in ex vivo models would also be interesting and would suggest indirect mechanisms. One such mechanism could be dysbiosis secondary to the effect of fumonisins on the intestinal microbiota [61,62,63]. Indeed, disturbance of the gut microbiota has been reported to induce changes in short-chain fatty acid levels and to have an impact on fatty liver disease and neuroinflammatory diseases [64,65].

## 5. Conclusions

In conclusion, fumonisins, even at a dose deemed safe, have a profound impact on the hepatic and brain oxylipidomes in chickens. In the liver, the effects of fumonisins were not dependent on the ω3- or ω6-character of the PUFAs but rather on the chain length, with a more pronounced increase in OLs derived from C18-PUFAs compared to those from C20 or C22 PUFAs. After four days of fumonisins feeding, a specific increase in OLs formed by the P450 pathway was observed, while at nine days, OLs from P450, along with several from the COX and LOX pathways, were also elevated. The diol/epoxide ratios were strongly reduced at both four and nine days of fumonisins exposure. In the brain, OLs derived from ω3-PUFAs were more indicative of fumonisins’ effects than those of ω6-PUFAs, particularly after four days of feeding the contaminated diet. The most markedly increased OLs were those enzymatically formed by the LOX and COX pathways, with a smaller increase from the P450 pathway. A variable rise in the diol/epoxide ratios was observed at both four and nine days. Collectively, these results suggest that the effects of fumonisins on OLs in the liver and brain are both specific and distinct. The changes in OLs in the liver are likely due to P450-mediated alterations and do not appear to be related to an inflammatory process, while the changes in the brain are indicative of an inflammatory/anti-inflammatory response, potentially linked to the effects of fumonisins on specialized cells, such as macrophages or glial cells. Further research is necessary to confirm these hypotheses.

## Figures and Tables

**Table 1:** Oxylipins in the liver of chickens fed a control diet free of mycotoxins (CONs) and chickens fed a diet containing 20.8 mg FB1 + FB2/kg for four days (FB_4d) or nine days (FB_9d).

Analyte	Fatty Acid ^1^	Enzyme ^2^	CONs	FB_4d	FB_9d
5-iPF2alpha-VI	AA;20;6	NE	214 ± 200	184 ± 77	318 ± 81
PGF2alpha	AA;20;6	COX	93 ± 57	61 ± 24	112 ± 54
TXB2	AA;20;6	COX	103 ± 45 ^a^	49 ± 24 ^b^	88 ± 38 ^a^
PGD2	AA;20;6	COX	205 ± 284 ^b^	148 ± 85 ^b^	393 ± 152 ^a^
PGA2	AA;20;6	COX	43 ± 45 ^b^	32 ± 22 ^b^	85 ± 23 ^a^
12-HHTrE	AA;20;6	COX	186 ± 95	184 ± 47	236 ± 67
PGE2	AA;20;6	COX	125 ± 148	82 ± 45	143 ± 66
8,15-DiHETE	AA;20;6	15LOX	5301 ± 5519 ^b^	3114 ± 1395 ^b^	8809 ± 3318 ^a^
5,15-DiHETE	AA;20;6	15LOX	364 ± 379 ^ab^	216 ± 109 ^b^	601 ± 231 ^a^
6-trans-LTB4	AA;20;6	LOX	68 ± 65 ^b^	50 ± 28 ^b^	137 ± 51 ^a^
Lipoxin-A4	AA;20;6	15LOXm	315 ± 371 ^ab^	257 ± 128 ^b^	650 ± 154 ^a^
15-HETE	AA;20;6	15LOX/P450	8929 ± 9849	4173 ± 1646	8201 ± 3881
5-HETE	AA;20;6	5LOX/P450	8816 ± 9014 ^ab^	3497 ± 868 ^b^	7106 ± 2458 ^a^
15-KETE	AA;20;6	15LOX	2376 ± 2092	1288 ± 438	1959 ± 720
5-KETE	AA;20;6	5LOX	1865 ± 1356	1315 ± 400	2348 ± 844
12-HETE	AA;20;6	LOX/P450	5283 ± 6147 ^ab^	2946 ± 1085 ^b^	6966 ± 2852 ^a^
11-HETE	AA;20;6	P450/NE	8464 ± 7338 ^ab^	3787 ± 1462 ^b^	8372 ± 2996 ^a^
20-HETE	AA;20;6	P450	40 ± 32 ^b^	82 ± 40 ^a^	92 ± 44 ^a^
9-HETE	AA;20;6	P450	3646 ± 4913 ^a^	581 ± 627 ^b^	956 ± 630 ^ab^
8-HETE	AA;20;6	P450	2000 ± 2630 ^ab^	1165 ± 333 ^b^	2370 ± 833 ^a^
14,15-EpETrE	AA;20;6	P450	499 ± 346 ^b^	959 ± 343 ^a^	1032 ± 384 ^a^
11,12-EpETrE	AA;20;6	P450	19 ± 28 ^b^	155 ± 91 ^a^	170 ± 96 ^a^
8,9-EpETrE	AA;20;6	P450	479 ± 495 ^c^	1700 ± 769 ^b^	2776 ± 880 ^a^
14,15-DiHETrE	AA;20;6	P450	728 ± 328	596 ± 193	795 ± 207
11,12-DiHETrE	AA;20;6	P450	678 ± 321 ^b^	684 ± 187 ^b^	1035 ± 272 ^a^
8,9-DiHETrE	AA;20;6	P450	1327 ± 662 ^ab^	1210 ± 431 ^b^	1857 ± 619 ^a^
5,6-DiHETrE	AA;20;6	P450	2765 ± 1617	2216 ± 842	3503 ± 1395
15-HETrE	DGLA;20;6	15LOX	1953 ± 2178 ^ab^	583 ± 358 ^b^	1832 ± 1101 ^a^
13-HODE	LA;18;6	15LOX	27,876 ± 28,043 ^b^	34,309 ± 22,461 ^b^	100,804 ± 58,526 ^a^
9-HODE	LA;18;6	LOX	2862 ± 3011	3295 ± 1939	9543 ± 8824
13-KODE	LA;18;6	15LOX	11,065 ± 14,730 ^b^	12,446 ± 6134 ^b^	33,705 ± 10,523 ^a^
9-KODE	LA;18;6	LOX	60,819 ± 40,501 ^c^	125,911 ± 78,249 ^b^	236,538 ± 13,1939 ^a^
9,12,13-TriHOME	LA;18;6	15LOX	2104 ± 1762 ^b^	3581 ± 1609 ^b^	8540 ± 2111 ^a^
9,10,13-TriHOME	LA;18;6	LOX	2969 ± 2593 ^b^	5767 ± 2980 ^b^	14,175 ± 6346 ^a^
12,13-EpOME	LA;18;6	P450	557 ± 196 ^c^	1994 ± 799 ^b^	2770 ± 866 ^a^
9,10-EpOME	LA;18;6	P450	1079 ± 490 ^c^	2579 ± 912 ^b^	3491 ± 985 ^a^
12,13-DiHOME	LA;18;6	P450	948 ± 369 ^c^	1383 ± 491 ^b^	2469 ± 777 ^a^
9,10-DiHOME	LA;18;6	P450	1542 ± 605 ^c^	2196 ± 692 ^bc^	4469 ± 1537 ^a^
5-HEPE	EPA;20;3	NE	112 ± 72	68 ± 14	90 ± 40
8-HEPE	EPA;20;3	NE	90 ± 87	53 ± 20	103 ± 53
11-HEPE	EPA;20;3	NE	111 ± 100	63 ± 26	138 ± 65
12-HEPE	EPA;20;3	NE	163 ± 147	99 ± 31	200 ± 85
18-HEPE	EPA;20;3	NE	318 ± 350 ^a^	181 ± 86 ^b^	477 ± 225 ^a^
15-HEPE	EPA;20;3	15LOX	124 ± 137 ^ab^	60 ± 31 ^b^	153 ± 92 ^a^
17,18-DiHETE	EPA;20;3	P450	30 ± 15 ^b^	34 ± 15 ^b^	48 ± 15 ^a^
14,15-DiHETE	EPA;20;3	P450	14 ± 10 ^ab^	11 ± 4 ^b^	20 ± 7 ^a^
11,12-DiHETE	EPA;20;3	P450	27 ± 14	24 ± 7	35 ± 10
17-oxo-DPA	DPA;22;3	LOX	590 ± 419	347 ± 146	551 ± 279
19,20-EpDPE	DHA;22;3	P450	196 ± 137	243 ± 74	306 ± 75
16,17-EpDPE	DHA;22;3	P450	137 ± 89 ^b^	252 ± 106 ^a^	258 ± 114 ^a^
19,20-DiHDPA	DHA;22;3	P450	108 ± 50	125 ± 51	153 ± 47
17-HDoHE	DHA;22;3	15LOX/NE	425 ± 321 ^ab^	228 ± 91 ^b^	471 ± 267 ^a^
14-HDoHE	DHA;22;3	15LOX/NE	556 ± 435	338 ± 175	532 ± 285
16-HDoHE	DHA;22;3	NE	473 ± 430 ^ab^	247 ± 111 ^b^	595 ± 325 ^a^
13-HDoHE	DHA;22;3	NE	728 ± 529	375 ± 145	714 ± 366
11-HDoHE	DHA;22;3	NE	383 ± 344	363 ± 137	550 ± 218
10-HDoHE	DHA;22;3	NE	271 ± 181	160 ± 53	259 ± 118
8-HDoHE	DHA;22;3	NE	585 ± 434 ^a^	225 ± 121 ^b^	306 ± 139 ^ab^
7-HDoHE	DHA;22;3	NE	91 ± 88	38 ± 22	43 ± 17
4-HDoHE	DHA;22;3	NE	525 ± 302	411 ± 113	581 ± 210
12,13-DiHODE	αLA;18;3	P450	1634 ± 715 ^b^	2553 ± 1290 ^b^	4487 ± 1625 ^a^
9,10-DiHODE	αLA;18;3	P450	79 ± 31 ^b^	128 ± 68 ^b^	228 ± 78 ^a^
9-HOTrE	αLA;18;3	5LOX	224 ± 218 ^b^	305 ± 144 ^b^	934 ± 442 ^a^
13-HOTrE	αLA;18;3	15LOX	71 ± 78 ^b^	90 ± 39 ^b^	288 ± 147 ^a^

^1^ AA;20;6 = arachidonic acid, C20:4, ω-6; DGLA;20;6 = dihomo-gamma-linoleic acid, C20:3, ω-6; LA;18;6 = linoleic acid, C18:2, ω-6; EPA;20;3 = eicosapentaenoic acid, C20:5, ω-3; DPA;22;3 = docosapentaenoic acid, C22:5, ω-3; DHA;22;3 = docosahexaenoic acid, C22:6, ω-3; αLA;18;3 = alpha-linolenic acid, C18:3, ω-3. ^2^ attributed according to [14]. Results are expressed in ng OL/g of liver as mean ± SD, n = 10. ANOVA was used to assess differences between groups. Statistically different groups (Duncan) are identified by different letters (*p* < 0.05).

**Table 2:** Oxylipins in the brain of chickens fed a control diet free of mycotoxins (CONs) and chickens fed a diet containing 20.8 mg FB1 + FB2/kg for four days (FB_4d) or nine days (FB_9d).

Analyte	Fatty Acid ^1^	Enzyme ^2^	Con	FB_4d	FB_9d
8-iso-PGF2α	AA;20;6	NE	34 ± 7 ^ab^	30 ± 10 ^b^	42 ± 10 ^a^
5-iPF2α-VI	AA;20;6	NE	86 ± 13 ^b^	83 ± 27 ^b^	116 ± 30 ^a^
PGF2α	AA;20;6	COX	106 ± 27 ^b^	85 ± 15 ^c^	133 ± 24 ^a^
TXB2	AA;20;6	COX	33 ± 9 ^b^	29 ± 5 ^b^	41 ± 6 ^a^
PGD2	AA;20;6	COX	21 ± 7	21 ± 8	26 ± 15
PGA2	AA;20;6	COX	14 ± 6	16 ± 6	20 ± 6
12-HHTrE	AA;20;6	COX	106 ± 21 ^b^	127 ± 15 ^a^	133 ± 23 ^a^
PGE2	AA;20;6	COX	12 ± 6	17 ± 8	15 ± 7
8,15-DiHETE	AA;20;6	15LOX	405 ± 125	511 ± 173	530 ± 161
5,15-DiHETE	AA;20;6	15LOX	27 ± 9	29 ± 15	39 ± 16
LTB4	AA;20;6	LOX	50 ± 10	49 ± 12	56 ± 14
6-trans-LTB4	AA;20;6	LOX	15 ± 4	15 ± 5	19 ± 4
Lipoxin-A4	AA;20;6	15LOX	29 ± 10	31 ± 12	36 ± 13
15-HETE	AA;20;6	15LOX/P450	499 ± 149	695 ± 236	721 ± 262
5-HETE	AA;20;6	5LOX/P450	659 ± 150	793 ± 220	890 ± 276
15-KETE	AA;20;6	15LOX	97 ± 68	90 ± 62	127 ± 82
5-KETE	AA;20;6	5LOX	162 ± 75	138 ± 50	198 ± 88
12-HETE	AA;20;6	LOX/P450	367 ± 128	505 ± 186	441 ± 164
11-HETE	AA;20;6	P450/NE	423 ± 115	568 ± 170	532 ± 195
9-HETE	AA;20;6	P450	133 ± 50	195 ± 79	155 ± 75
8-HETE	AA;20;6	P450	139 ± 47	174 ± 55	153 ± 38
14,15-EpETrE	AA;20;6	P450	31 ± 20	45 ± 25	22 ± 16
11,12-EpETrE	AA;20;6	P450	6.9 ± 5 ^ab^	10 ± 4 ^a^	5 ± 3 ^b^
8,9-EpETrE	AA;20;6	P450	464 ± 196 ^a^	298 ± 134 ^b^	498 ± 145 ^a^
14,15-DiHETrE	AA;20;6	P450	55 ± 12	52 ± 11	60 ± 17
11,12-DiHETrE	AA;20;6	P450	59 ± 15	55 ± 12	63 ± 20
8,9-DiHETrE	AA;20;6	P450	76 ± 21	70 ± 15	84 ± 27
5,6-DiHETrE	AA;20;6	P450	159 ± 58	146 ± 36	170 ± 53
PGF1α	DGLA;20;6	COX	20 ± 3	19 ± 4	22 ± 5
15-HETrE	DGLA;20;6	15LOX	72 ± 19	92 ± 28	93 ± 26
13-HODE	LA;18;6	15LOX	193 ± 118	170 ± 54	259 ± 136
9-HODE	LA;18;6	LOX	33 ± 22	31 ± 10	44 ± 20
13-KODE	LA;18;6	15LOX	47 ± 22	39 ± 24	59 ± 28
9-KODE	LA;18;6	LOX	850 ± 535	611 ± 452	1119 ± 633
9,12,13-TriHOME	LA;18;6	15LOX	158 ± 43	121 ± 26	192 ± 75
9,10,13-TriHOME	LA;18;6	LOX	185 ± 47	143 ± 28	225 ± 81
9,10-EpOME	LA;18;6	P450	9.1 ± 5	14 ± 13	8 ± 5
12,13-DiHOME	LA;18;6	P450	13 ± 4	16 ± 7	15 ± 6
9,10-DiHOME	LA;18;6	P450	14 ± 5	18 ± 8	19 ± 9
5-HEPE	EPA;20;3	NE	2.5 ± 1	3 ± 1	5 ± 3
11-HEPE	EPA;20;3	NE	1.5 ± 0 ^b^	2 ± 1 ^ab^	3 ± 1 ^a^
17-oxo-DPA	DPA;22;3	LOX	110 ± 32 ^b^	206 ± 58 ^a^	171 ± 54 ^a^
16,17-EpDPE	DHA;22;3	P450	14 ± 9 ^b^	32 ± 15 ^a^	12 ± 8 ^b^
19,20-EpDPE	DHA;22;3	P450	86 ± 22 ^b^	127 ± 24 ^a^	122 ± 25 ^a^
19,20-DiHDPA	DHA;22;3	P450	22 ± 5	23 ± 4	26 ± 6
10,17-DiHDoHE	DHA;22;3	LOX	6.4 ± 4	9 ± 5	8 ± 3
17-HDoHE	DHA;22;3	15LOX/NE	102 ± 27 ^b^	159 ± 45 ^a^	146 ± 51 ^a^
16-HDoHE	DHA;22;3	NE	113 ± 35 ^ab^	186 ± 64 ^b^	176 ± 60 ^a^
14-HDoHE	DHA;22;3	12,15LOX/NE	114 ± 34 ^b^	199 ± 48 ^a^	172 ± 56 ^a^
13-HDoHE	DHA;22;3	NE	133 ± 25 ^b^	214 ± 45 ^a^	199 ± 56 ^a^
11-HDoHE	DHA;22;3	NE	103 ± 47 ^b^	172 ± 51 ^a^	143 ± 41 ^ab^
10-HDoHE	DHA;22;3	NE	48 ± 19 ^b^	88 ± 26 ^a^	74 ± 23 ^a^
8-HDoHE	DHA;22;3	NE	110 ± 33 ^c^	207 ± 51 ^a^	153 ± 41 ^b^
7-HDoHE	DHA;22;3	NE	29 ± 9 ^b^	48 ± 10 ^a^	34 ± 8 ^b^
4-HDoHE	DHA;22;3	NE	337 ± 76 ^b^	481 ± 110 ^a^	518 ± 94 ^a^
9,10-DiHODE	αLA;18;3	P450	0.9 ± 1	2 ± 1	2 ± 2
9-HOTrE	αLA;18;3	5LOX	1 ± 1	1 ± 1	3 ± 3
13-HOTrE	αLA;18;3	15LOX	3.8 ± 3	5 ± 3	8 ± 7

^1^ AA;20;6 = arachidonic acid, C20:4, ω-6; DGLA;20;6 = dihomo-gamma-linoleic acid, C20:3, ω-6; LA;18;6 = linoleic acid, C18:2, ω-6; EPA;20;3 = eicosapentaenoic acid, C20:5, ω-3; DPA;22;3 = docosapentaenoic acid, C22:5, ω-3; DHA;22;3 = docosahexaenoic acid, C22:6, ω-3; αLA;18;3 = alpha-linolenic acid, C18:3, ω-3. ^2^ attributed according to [14]. Results are expressed in ng OL/g of brain as mean ± SD, n = 10. ANOVA was used to assess differences between groups. Statistically different groups (Duncan) are identified by different letters (*p* < 0.05).

## Data Availability

All the data presented are provided in the manuscript and as a supplementary file. Individual data may be supplied on reasoned request.

## Supplementary Materials

The following supporting information can be downloaded at: https://www.mdpi.com/article/10.3390/antiox14010019/s1, Table S1: MRM parameters and retention times of oxylipins quantified in this study; Table S2: Stability of oxylipins measured at different concentrations after 8, 16, 24, and 32 h of storage at room temperature; Table S3: Linearity of the method of analysis; Table S4: Recovery of oxylipins measured after solubilization in saline phosphate buffer; Table S5: Signal suppression and enhancement measured on the internal standards (ISs) of oxylipins in the liver and brain of chickens; Table S6: Recovery of oxylipins measured for the internal standards (ISs); Table S7: Intra- and inter-day repeatability measured on the internal standards (ISs).
